# Hofmeister Anion-Induced Tunable Rheology of Self-Healing Supramolecular Hydrogels

**DOI:** 10.1186/s11671-018-2823-8

**Published:** 2019-01-07

**Authors:** Jing Zhang, Baohao Zhang, Qiang Chen, Bao Zhang, Jian Song

**Affiliations:** 1 School of Chemical Engineering and Technology, Tianjin University, Tianjin, 300350 China; 2 The Co-Innovation Center of Chemistry and Chemical Engineering of Tianjin, Tianjin, 300072 China; 3 Renai College of Tianjin University, Tianjin, 301636 China

**Keywords:** Gelator, Rheology, Self-assembly, Self-healing, Thixotropy

## Abstract

**ᅟ:**

Physical gelation behaviors of a series of d-gluconic acetal-based derivatives bearing fatty alkyl amine moieties have been investigated. One of these molecules exhibits excellent gelation behaviors in water, and the resultant hydrogels are found to display self-healing properties. Interestingly, the elasticity and strength of the resulting gel can be tuned by the addition of different kinds of Hofmeister salts. The gel formation mechanism was proposed based on the analysis of FT-IR,^1^HNMR, and XRD, indicating that the main driving force for the self-assembly was the π-π stacking of the benzene rings in the aqueous solution system. Overall, our research provides an efficient approach for facilely tuning the properties of the d-gluconic acetal-based hydrogel.

**ᅟ:**

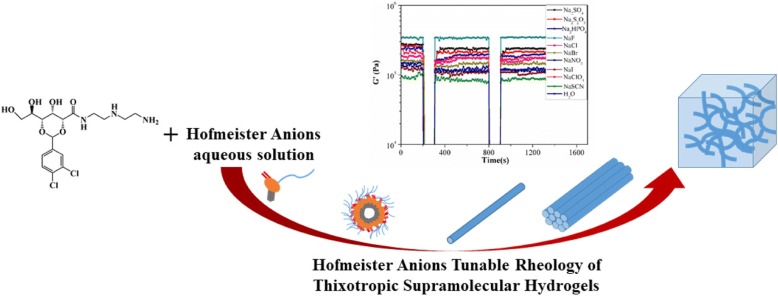

**Electronic supplementary material:**

The online version of this article (10.1186/s11671-018-2823-8) contains supplementary material, which is available to authorized users.

## Background

Gels composed of low molecular mass gelators (LMMGs) can be obtained from the self-assembly of LMMGs via supramolecular interactions such as hydrogen bonding, van der Waals interactions, π-π stacking, and so on [1–13]. Over the last few decades, there has been rapid progress in the synthesis of LMMGs and supramolecular gels derived from LMMGs owing to the dynamic interaction of the gelators. They have attracted much attention not only as alternative materials for polymer gels but also as new types of soft materials capable of responding to multiple external stimuli. Furthermore, self-healing, an ability of systems to spontaneously repair their damage and improve the life-time of materials [14–24], is one of the most fascinating properties of materials. They have found potential applications in the fields such as biological tissue [25], shape memory [24], and sensors [14–24] as advanced materials. The development of self-healable supramolecular gels is one of the emerging fields in materials research. However, the reported self-healing gels are mainly polymeric gels [25–31]. Even though they have enhanced mechanical properties, these synthetic polymer-based hydrogels do not have cell compatibility or degradability, which are critical for emerging biomedical applications [32–34]. Low molecular weight gelators (LMWGs)-based self-healing organogels [35–39] and hydrogels have been recently reported. To enhance the application of the resulting gels, efforts to develop molecular gels with tunable rheological properties are of pivotal importance, which will also provide new insights into the relationship between the molecular structure of gelators and gelation ability. In general, rheological behaviors of molecular gels can be modulated by introducing a new component into the original gel systems, such as metal complexes, neutral molecules, and inorganic salts [40–45].

**Fig. 1:**
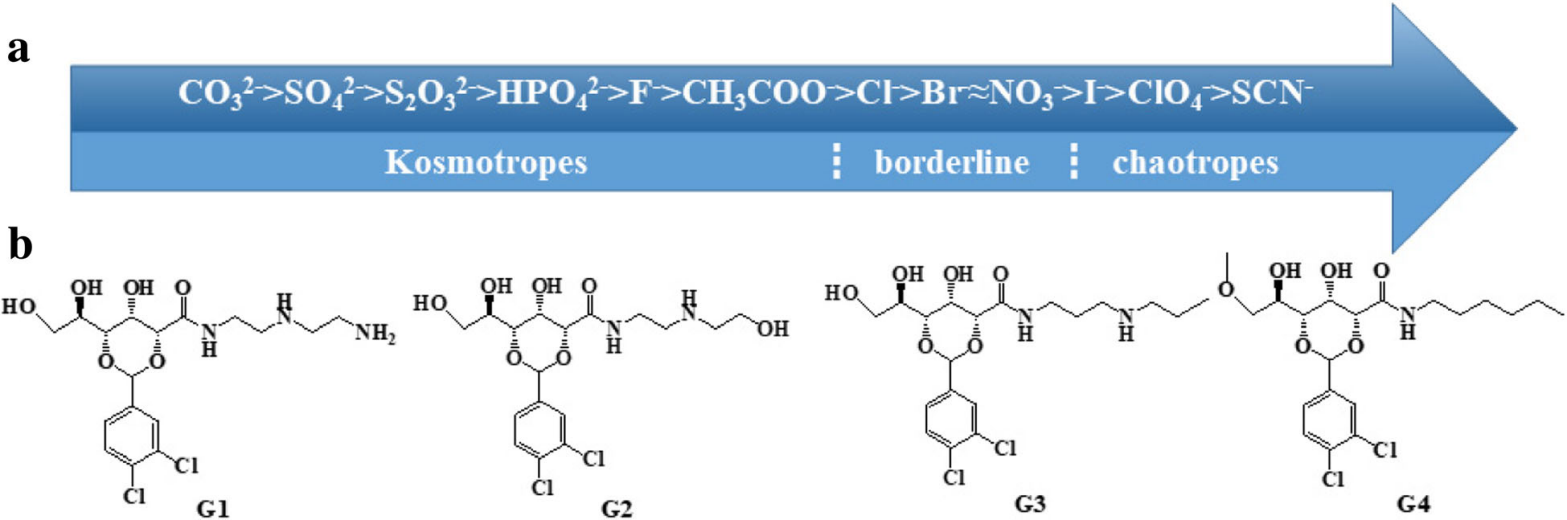
**a** Hofmeister anions. **b** Chemical structures of gelator Gn (*n* = 1–4) examined

Although Hofmeister described the “specific ion” effect on protein folding and aggregation in 1888, the understanding of this effect still remains controversial [46, 47]. Hofmeister classified the ions according to their relative ability of stabilizing protein in aqueous solutions, resulting in the sequence shown in Fig. 1a. The ions on the left side of the series are kosmotropes (well-hydrated ions), and those on the right side are chaotropes (poorly hydrated ions). The former decreases and the latter increases protein solubility. The molecular basis of such phenomena has long been attributed to changes of structures in the water induced by salts [48]. However, recent evidence has suggested that the direct interactions between the ions and the macromolecules and their hydration shells result in the removal of the hydratation water molecules of the proteins, leading to their folding and precipitation, which could be the reason for the Hofmeister effect [49–52]. These specific ion effects have later been observed in other areas, including colloid and surface chemistry, and macromolecular systems, such as proteins and polymers, etc. [53–60]. Due to the strong effect on macromolecules dissolved in water, salts have also been used to monitor the properties of hydrogels [61–68]. Rowan et al. [53] reported that they used the Hofmeister effect to controllably manipulate the mechanical properties of ethylene glycol-functionalized polyisocyanides-based polymer hydrogels. Wang et al. [69–71] demonstrated that they produced hydrogels with high ductility, strength, recoverability, and fatigue resistance without modifying the gelatin chains or adding chemical crosslinks or fillers by soaking gelatin hydrogels in (NH_4_)_2_SO_4_ solutions at appropriate concentrations. However, it has only recently been appreciated in supramolecular chemistry and, more specifically, LWMGs-based hydrogels. For example, Mocerino et al. [61–68] produced a macrocyclic low molecular weight hydrogelator, proline-functionalized calix [25–31] arene, which showed ion-specific sol–gel transition, but the effect of the salts on the molecular organization of the hydrogel was not studied. Roy et al. [61] demonstrated that salts have the ability to regulate the morphology, material stiffness, and chirality properties of supramolecular hydrogels using hydrophobic peptide-based hydrogelators. Then, Roy et al. [66] reported that salts have a dramatic effect on the molecular self-assembly of the aromatic dipeptide amphiphiles in aqueous media. Salts affect the structure of the enzyme network, which, in turn, influence the enzyme kinetics and the corresponding nucleation and growth of the nanostructures. Escuder et al. [61–68] prepared smart supramolecular hydrogels from a bolaamphiphilic l-valine derivative in aqueous solutions of different salts. The hydrogels selectively responded to different ions and strengths of them were either reinforced or weakened. Recently, our group reported a multifunctional d-gluconic acetal-based gelator with a long alkyl chain which could form gels with outstanding self-healing ability [72, 73]. We also reported the multifunctional gel systems based on the amine-acid two-component systems which showed high-efficiency self-healing, room-temperature phase selective gelation, and dye removal capabilities [74]. Based on these works, here we designed a series of organic compounds (Gn, *n* = 1–4, Fig. 1b) derived from d-gluconical bearing alkyl amine moieties. These compounds were highly efficient LMWGs based on hydrogen-bonding interaction and π-π stacking force. Hydrogen bonding was one of the main self-assembly driving forces of supramolecular gels. By introducing an amino or hydroxyl in alkyl chain, or substituting the active hydrogen of hydroxy by methyl iodide, hydrogen bonding sites were added or adjusted. The compounds were serving as novel and highly efficient LMWGs (cf. Fig. 1b).

Initially, the gelation abilities of Gn in various solvents were summarized in Additional file 1: Table S1. It was found that Gn were potent gelators in certain organic solvents. Except for G3 and G4, the others have the abilities to gelatinize water. In particular, G1-gel was found to have self-healing properties during the test. The G1 gel can undergo an instant self-healing process upon mechanical damage. Furthermore, we explored the effect of salts on the properties of the gels, especially the recovery properties.

To the best of our knowledge, few reports have been disclosed with tunable rheology of supramolecular gels by addition of a slight amount of Hofmeister salts.

## Methods/Experimental

### Materials

d-Gluconic acid, 3, 4-dichlorobenzaldehyde, β-hydroxyethylenediamine, 2, 2-iminodi (ethylamine),1-aminohexane, and *N*-propyl-propane-1,3-diamine were purchased from Shanghai Jingchen Scientifical Co., Ltd. The chemical reagents were commercially available and directly utilized without further purification. 2,4-(3, 4-Dichloro) benzylidene methy-d-gluconate was synthesized by the methods reported previously [75]. Characterizations of a new compound Gn are provided in Additional file 1. Synthetic routes of Gn are shown in Additional file 1: Scheme S1. The detailed synthetic procedures and characterization data of Gn are given in Additional file 1.

### Gel Preparation

Gelation tests for Gn in organic solvents were investigated by a typical tube inversion method. Gn were mixed in an organic solvent (1 mL) in a sealed test tube, which was heated until the solid was completely dissolved and then cooled to room temperature. Finally, the test tube was inversed to observe whether the solution inside could still flow [69–71]. Gelation was considered to have occurred when a homogeneous substance was obtained which exhibited no gravitational flow, and it was denoted by “G.” Solution and solid-like gel may coexist within a system as “partial gels (PG)”. Systems, in which only solution was obtained, were referred to as solution (S). If clear solutions were obtained when they are hot, but precipitation occurs when they are cooled down to room temperature, these systems are denoted by “precipitation (P).” In an insoluble system (I), gelator could not be dissolved even at the boiling point of the solvent. The critical gelation concentrations (CGCs) means the minimum amount of gelator required to immobilize 1 mL of solvent.

### ^1^H NMR

^1^H NMR spectroscopic measurements were carried out on Bruker DPX 400 MHz spectrometer. In the temperature experiments, 10 mg of gelators was dissolved in 0.5 mL of D_2_O-d6. The temperature was 25 °C.

### Preparation of “Co-Gels” for Salt-Effects on Gel Rheology

A total of 20 mg gelator was added into 1.0 mL water. The resulting mixture was heated until it formed a clear solution and allowed to cool down to ambient temperature to form gels. For comparison, a reference gel was prepared only by introducing 20 mg gelator into 1.0 mL 0.5 M salt solution.

### Field Emission Scanning Electron Microscope

The morphologies of the xerogels were obtained by a Hitachi S-4800 SEM instrument operating at 3–5 kV. Samples were prepared by dropping the diluted solution of gels on the thin aluminum sheets and then dried under vacuum for 24 h. The samples were coated with a thin layer of Au before the experiment.

### FT-IR Spectroscopy

IR spectra were collected by a FTS3000 spectrometer with KBr pellets. The xerogels were prepared by drying hydrogels on glass slides under vacuum for 24 h.

### Powder X-Ray Diffraction

Powder X-ray diffraction (PXRD) diagrams of xerogels which were prepared from hydrogels were obtained by using a Bruker D8-S4 (CuKα radiation, λ = 1.546 Å). The d spacing values were calculated by Bragg’s law (nλ = 2d sinθ).

### Rheology Measurements

Rheology experiments were carried out with a strain-controlled rheometer (Anton Paar Physica MCR 301) equipped with steel-coated parallel-plate geometry (15 mm diameter). The gap distance was fixed at 0.5 mm. A solvent trapping device was placed above the plate and measurement was set at 20 °C in order to avoid solvent evaporation. The frequency sweep at a constant strain of 0.1% was obtained from 0.1 to 100 rad s^−1^. Strain sweep was performed in the 0.01–1000% range at a constant frequency (1 Hz). The time sweep was conducted to observe the recovery property of the gel. First, a constant strain of 0.1% was applied on the sample. Then a constant strain of 100% was applied to destroy the sample. And then a constant strain (0.1%) was applied again. The storage modulus G′ and the loss modulus G′′ of the sample were monitored as functions of time in this experiment.

## Results and Discussion

### Specific Ion Effects on the Solution-to-Gel Equilibrium

The gelation abilities of Gn are summarized in Additional file 1: Table S1. Fifteen different solvents were employed to evaluate their gelation behaviors. Compounds G3 and G4 could not gelatinize water. The hydrogel of G2 exhibits weakly.The CGCs (a minimum gel concentration) of hydrogel based on G1 is 25 mM. Here, compound G1 was chosen for the further study of the Hofmeister effect on the hydrogelation behavior.

The anions (as their sodium salts) employed in this study covered all of the relative positions in the Hofmeister series: kosmotropes (SO_4_^2−^, S_2_SO_3_^2−^, HPO_4_^2−^, F^−^), borderline (Br^−^, Cl^−^, NO_3_^−^), and chaotropes (I^−^, ClO_4_^−^,SCN^−^).

CGC measurements (“Gel Preparation” section) were carried out in order to investigate the effect of a series of salts on the gelator’s gelation ability. As shown in Additional file 1: Table S2, the CGCs of G1 in water increased gradually, by adding salt ions (0.5 M), from the kosmotropes ions (salt-out ions, e.g., SO_4_^2−^) to the chaotropes ions (salt-in ions, such as the SCN^−^).

**Fig. 2:**
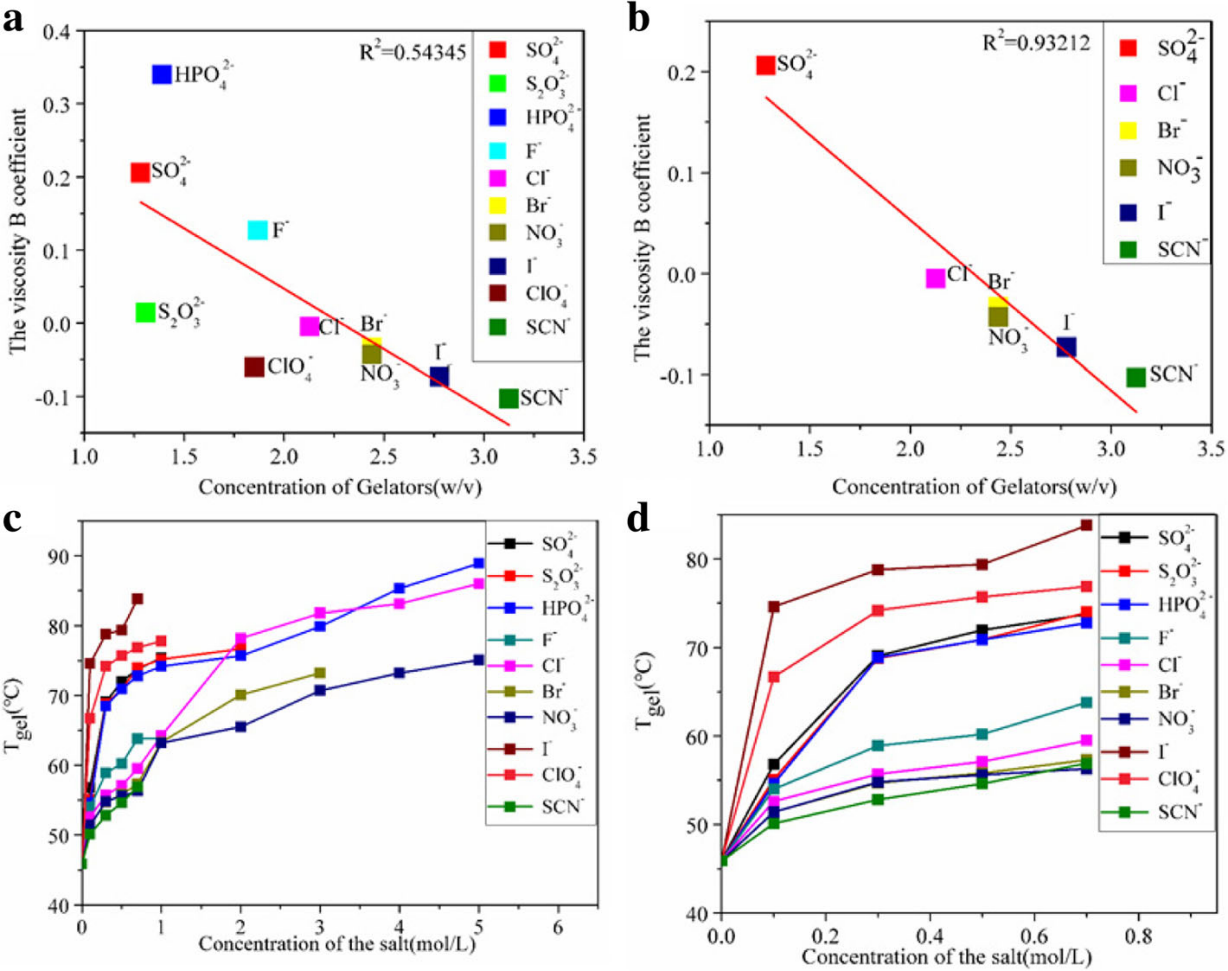
**a** CGCs (0.5 M salts) with the viscosity B coefficients. **b** CGCs (0.5 M salts) except the special points with the viscosity B coefficients. **c** Plot of the effect of anions (the salt concentration from 0 to 5 M) and T_gel_ of G1 hydrogel as a function of salt concentration. **d** Plot of the effect of anions (the salt concentration from 0 to 0.7 M) and T_gel_ of G1 hydrogel as a function of salt concentration

Because the higher the viscosity B coefficient of an anion is, the stronger it makes hydrogen bonds with water, by studying the correlation between the viscosity B coefficient of different anions and CGCs, the influence of different anions on the viscosity B coefficient of aqueous solution can be characterized [75] The fitting result was 0.543 (In Fig. 2a). When removed the certain points (ClO_4_^−^, F^−^, S_2_O_3_^2−^), the result was fitting as high as 0.932 (In Fig. 2b). The viscosity B coefficients are increasing from chaotropes ions to kosmotropes ions. The kosmotropes ions (such as SO_4_^2−^) can improve the hydrogen bonding between water molecules and decrease the number of free water, resulting in the decline of the interaction between the gelator and water,which promoted precipitation and self-assembly of gelators. Therefore, the CGCs decreased. The effect of chaotropes ions (such as SCN^−^) is opposite. The existence of irregular ions (ClO_4_^−^, F^−^, S_2_O_3_^2−^) indicated that the effect of salt ions on the properties of hydrogels could not be fitted by a single parameter (the viscosity B coefficients).

T_gel_ is another important parameter to evaluate the gelation abilities of gelator in various solvents. The higher T_gel_ means the better thermal stability performance. Figure 2c, d reveals that the types and concentration of anions have strong influence on T_gel_ of G1 hydrogels (see the detailed data in the Additional file 1: Table S3). Except for I^−^and ClO_4_^−^, the T_gel_ of G1 hydrogel at certain salt concentration (for example salt concentration is 0.5 M) decreased gradually from kosmotropes ions to chaotropes ions. It is well known that Kosmotropes anions cancause the “salting-out” effect in proteins and reduce the solubility of the gelators resulting in the formation of the solid-like fibrillar network. Accordingly, “salting-in” or chaotropic anions produced a solubilization effect on the gelator network. In other words, kosmotropic anions strengthen the fibrillar network, whereas chaotropic anions produce a weakening effect on the hydrogel. Naturally, T_gel_ shows the decreasing trend from kosmotropes ions to chaotropes ions. As shown in Fig. 2d when increasing the concentration of all anions from 0 to 1 M, the T_gel_ of G1 hydrogel gradually increased. It may be that upon addition of salt ions, the 3D network structure of G1 hydrogel becomes dense, which raises T_gel_ of G1 hydrogel.

### Salt-Induced Tunable Rheology of Self-Healing Supramolecular Hydrogels

**Fig. 3:**
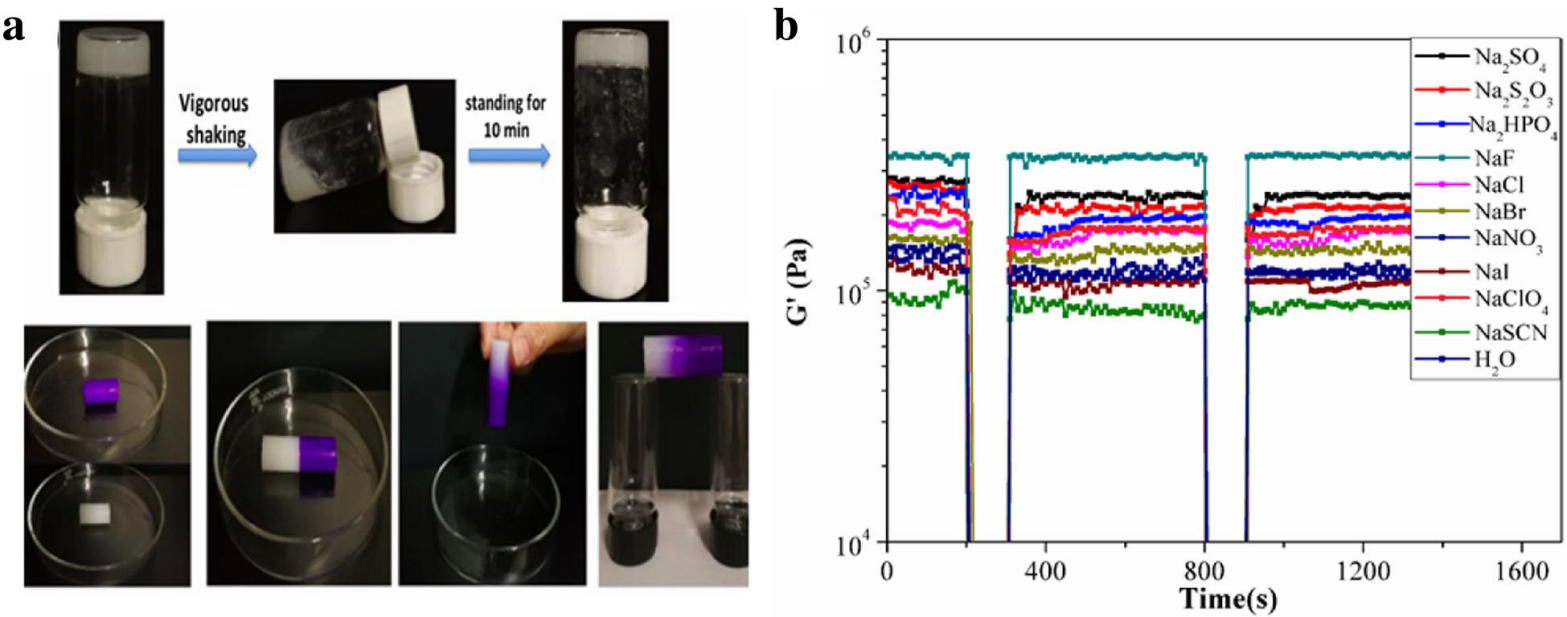
**a** Illustration of self-healing properties of the G1 gels (2.0% *w*/*v*) obtained from undoped gel and blue dye-doped gel. **b** Time scan tests under alternating strain of 0.1% and 100% of G1 (2.5% *w*/*v*) with Hofmeister salts (concentration is 0.5 M) with a fixed frequency at 1 Hz at 25 °C

As shown in Fig. 3a, upon shaking, liquid appeared leading to the sol-gel mixture. After resting for less than 10 min, the gel recovered. The self-healing properties of G1 gels were further demonstrated by cutting the gels into two pieces and then putting these pieces together. It was shown that the pieces joined together and merged into a continuous gel block (Fig. 3a). Next, we then explored the effect of salts on the the recovery properties of the gels.

The rheological behaviors of the supramolecular gels are important for their real-life applications, in particular, thixotropy and self-healing. In this section, to explore the effect of the Hofmeister salts on the mechanical and thixotropic behaviors of G1 hydrogels, the rheological properties of the G1 hydrogels(2.5% *w*/*v*) containing the a series of Hofmeister salts (salt concentration is 0.5 M) were examined.

When the frequency varied,the Additional file 1: Figure S2a revealed that Hofmeister anions can regulate the G′ value from 110,000 to 350,000 Pa and the range of the G′ value was 68.57%. Except for certain anions (F^−^and ClO_4_^−^), the G′ value decreased gradually from kosmotropes to chaotropes. Similarly, the Hofmeister anions can turn the G′′ value from 11,100 to 65,100 Pa and the range was 82.95%. The loss modulus(the G′ value) is a parameter reflecting the viscosity of the materials. The relationship between the loss modulus and Hofmeister salts was consistent with that between the viscosity B coefficients and Hofmeister salts. Figure 3b revealed that Hofmeister anions could regulate the recovered G′ value from 95,600 to 340,000 Pa. The range of the G′ value was 71.88%. Except for the specific anions (F^−^), the G′ value decreased gradually from kosmotropes to chaotropes. Besides, the step-strain measurement showed the recovery ratios of G′ after the first cycle (90.74%, 85.93%, 82.08%, 100%, 90.77%, 100%, 95.56%, 96.48%, 95.97%, 88.12%, 93.89% respectively), and these results illustrated that G1 hydrogel was of excellent thixotropic and self-healing properties.

To my best knowledge, this is the first time to regulate the thixotropy of hydrogels by using the Hofmeister anions. It also provides a simple and feasible method to regulate thixotropy.

### FT-IR Spectroscopy

**Fig. 4:**
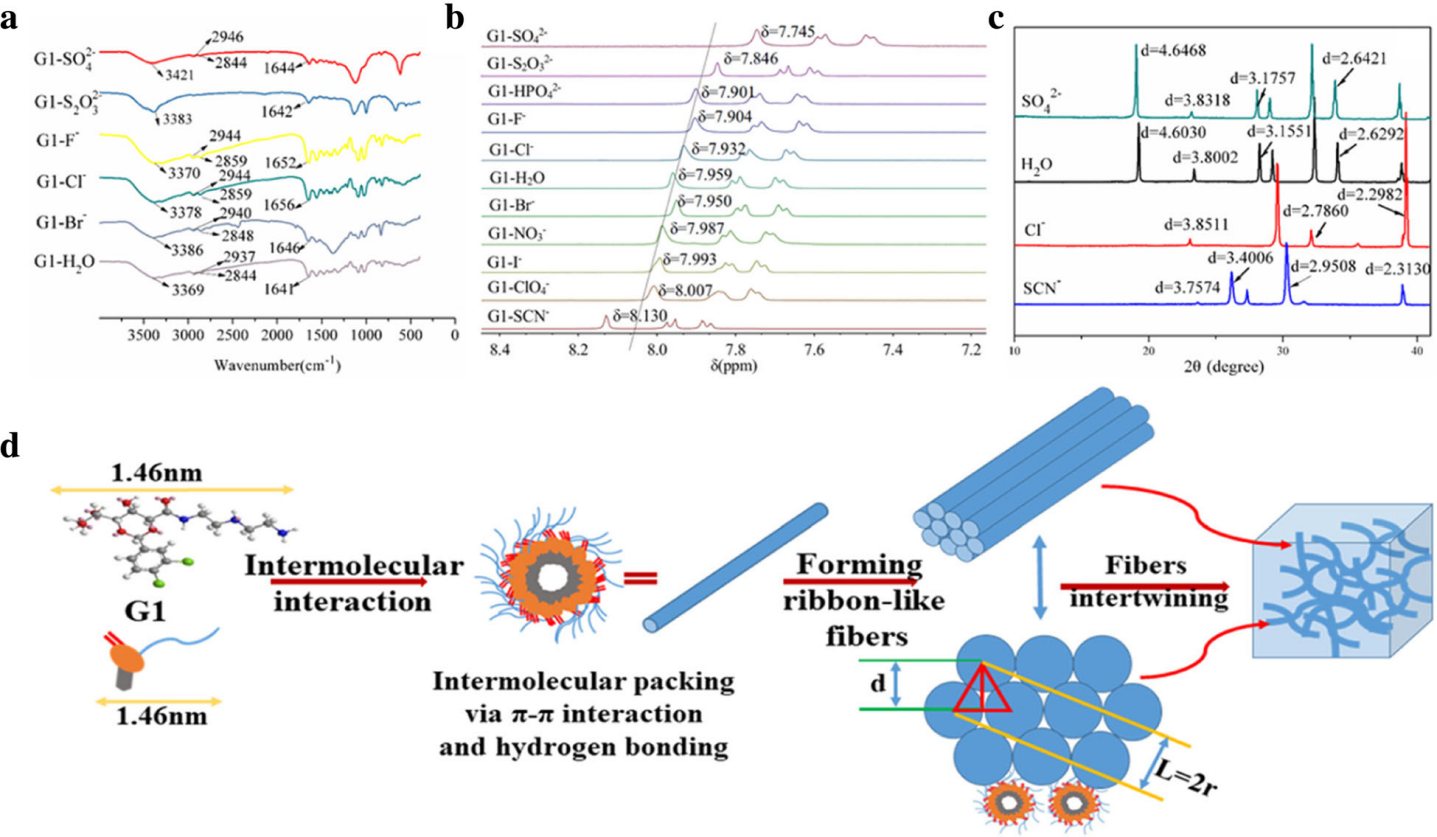
**a** FT-IR spectra of G1 xerogel (2.5% *w*/*v*) in the presence of some kosmotropes ions (concentration is 0.5 M). **b** ^1^H NMR spectra of G1 (2.5% *w*/*v*) in D_2_O addition of Hofmeister salts (concentration is 0.5 M). **c** WXRD spectra of 2.5% G1 xerogel obtained from water in the presence of Hofmeister anions (concentration is 0.5 M). **d** Possible self-assembly mechanism of G1 hydrogel with a schematic representation of the postulated molecular packing model (a: energy-minimized structure of G1) l

FT-IR was one of the effective methods to study the driving force of gels. FT-IR of G1 hydrogel (2.5% *w*/*v*) adding different salt solutions (0.5 M)were carried out and the results were shown in the Fig. 4a. The Fig. 4a of G1 xerogel revealed that about 2937 and 2844 cm^−1^ respectively, were characteristic for asymmetric stretching vibration (vas) and symmetric stretching vibration (vs) of methylene. With the addition of SO_4_^2−^, the corresponding bands were observed at 2946 and 2844 cm^−1^. This observation supported the weaker Van der Waals (VDW) interactions between the alkyl chains in the presence of the anions. In addition, the stretching band of O–H overlapped with that of N–H and appeard at 3369 cm^−1^. The stretching band of C=O was observed at 1640 cm^−1^. For the G1-SO_4_^2−^ and G1-S_2_O_3_^2−^ xerogel, the corresponding bands were observed at 3421, 1644 cm^−1^ and 3383, 1642 cm^−1^ respectively which were blue-shifted in contrasted with the G1 hydrogel. The xerogel of other anions showed similar trend. These results indicated that O–H, N–H, and C=O were involved in hydrogen bonding and the hydrogen bonding of hydrogel weakened because of the addition of kosmotropes anions of Hofmeister series. However, the thermodynamic stability reflected from T_gel_ and gelation capacity reflected from CGC were gradually getting better with the addition of kosmotropes anions. It was suggesting that in the self-assembly process, the hydrogen bonding was less than other forces and it was not the main driving force of self-assembly.

### ^1^H NMR

In fact, π-π interaction was recognized as one of the main driving force for the physical gelation of gelator G1 in the water as further evidenced by ^1^H NMR measurements (cf. Fig. 4b).

1H NMR spectra of the gelator G1 (2.5% *w*/*v*) with/without Hofmeister salts in D_2_O (salt concentration is 0.5 M) were compared, as shown in Fig. 4b. In Fig. 4b, one of the H-shifts on the benzene ring of G1 hydrogel appeared at 7.959 ppm in pure [d6]D_2_O. After the addition of SO_4_^2−^, S_2_O_3_^2−^, HPO_4_^2−^, and F^−^, the peaks of C–H protons shifted to 7.745, 7.846, 7.901, and 7.904 ppm, respectively. Compared with G1 in pure [d6]D_2_O, the signals of C–H protons on the benzene ring of the G1 mixed with kosmotropes anions (SO_4_^2−^, S_2_O_3_^2−^, HPO_4_^2−^, and F^−^) moved upfield, which indicated that the π-π stacking effects between benzene rings were enhanced. Correspondingly after the addition of I^−^, ClO_4_^2−^, and SCN^−^, the peaks of C–H protons shifted to 7.993, 8.007, and 8.130 ppm, respectively. Compared with G1 in pure [d6]D_2_O, the signals of C–H protons on the benzene ring of the G1 mixed with chaotropes anions (I^−^, ClO_4_^2−^, and SCN^−^) moved downfield, which suggested that the π-π stacking effects between benzene rings were weakened. In Fig. 4b with the addition of transition region anions (Cl^−^, Br^−^, and NO_3_^−^), the signals of C–H protons on the benzene ring shifted slightly, indicating that the π-π stacking effects between benzene rings were not influenced obviously.

From kosmotropes to chaotropes, H-shift on the benzene ring gradually moved toward the low field and the π-π stacking effect between benzene rings was gradually weakened*.* The result was consistent with the macro performance of the gelators (the changing trends of CGC and T_gel_). It also reveals that the main driving force for the self-assembly is the π-π stacking force of the benzene rings in the aqueous solution system.

### SEM

The morphologies of some typical gels were examined by SEM. As shown in Additional file 1: Figure S3a, ropelike left-handed helical structures with a pitch of about 50 nm are observed for G1 hydrogels. Ropelike left-handed helical fibers with a 30 nm average diameter that formed a 3D network were observed in aqueous solution of SO_4_^2−^ (concentration is 0.5 M, Additional file 1: Figure S3b). In addition, G1 hydrogels are composed of complex and entangled three-dimensional networks, and the fiber bundles are thick and dense. However, it can be seen that due to the salt-out effect of SO_4_^2−^, the gelator is precipitated out and wound into a more dense three-dimensional network structure. But due to the influence of Cl^−^ which belongs to the transition region of kosmotropes and chaotropes on the solubility, the gelators self-assemble into a mesh of fiber structure (Additional file 1: Figure S3c). In the image of hydrogels formed in the presence of the salt-in anion SCN^−^, it is shown that the fibers were broken and folded loosely together without the formation of the three-dimensional structure, so that the ability to bind solvents is relatively weak (Additional file 1: Figure S3d). Obviously, the morphologies and thermodynamic stability of the gel showed the same trend. In other words, the aggregation of gelators decreases from kosmotropes and chaotropes.

### Powder X-Ray Diffraction

To explore the possible packing modes of the gelator molecules in water with the addition of Hofmeister salts in particular, wide-angle XRD (WXRD) of the G1 xerogels were examined. As shown in Fig. 4c, XRD patterns of the G1 xerogel from water showed four main diffraction peaks centered at 2θ = 19.267 (*d* = 0.46030 nm), 2θ = 28.262 (*d* = 0.31551 nm), 2θ = 34.071 (*d* = 0.26292 nm), and 2θ = 38.843 (*d* = 0.23165), and the ratio of *d*-spacing values is about 1:1/√2:1:√3 indicating that the self-assembly of the G1 xerogel from water is composed of hexagonal closs packing possibly [76]. In addition, there was a main raction peak centered at 2θ = 23.389 (*d* = 0.38002 nm), and *d* = 0.38 nm is the characteristic of π-π stacking force of the benzene rings. It reveals that the main driving force for the self-assembly is the π-π stacking force of the benzene rings in the aqueous solution system [77, 78]. In Fig. 4c, patterns of the G1 xerogel from Na_2_SO_4_ aqueous solution (concentration is 0.5 M) exhibited four diffraction peaks centered at 2θ = 19.084 (*d* = 0.46468 nm), 2θ = 28.075 (*d* = 0.31757 nm), 2θ = 33.901 (*d* = 0.26421 nm), and 2θ = 38.683 (*d* = 0.23257 nm), and it proved that the addition of SO_4_^2−^ did not affect packing modes of the gelator molecules.While patterns of the G1 xerogel from NaSCN aqueous solution (concentration is 0.5 M) exhibited diffraction peaks centered at 2θ = 26.184 (*d* = 0.34006 nm), 2θ = 30.263 (*d* = 0.29508 nm), 2θ = 38.904 (*d* = 0.23130 nm), and 2θ = 38.683 (*d* = 0.23257 nm), and patterns of the G1 xerogel from aqueous solution (salt concentration is 0.5 M) exhibited four diffraction peaks centered 2θ = 23.076 (*d* = 0.38511 nm), 2θ = 29.563 (*d* = 0.30191 nm), 2θ = 32.101 (*d* = 0.27860 nm), and 2θ = 39.165 (*d* = 0.22982 nm). The ratio of *d*-spacing values were all 1:1/√2:1:√3 indicating that the self-assembly of the G1 xerogel from water is mainly composed of hexagonal closs packing [76]. At the same time, except the above-mentioned main peaks, other peaks also appear in the Fig. 4c, indicating that other packing modes may also exist in self-assembly process. The above results indicate that the addition of different anions had great influence on the properties of gels but it did not change the packing mode of the hydrogel self-assembly process.

### The Gel Formation Mechanism

Furthermore, the energy-minimized structure of G1 (Additional file 1: Figure S4) suggests that the length of the molecule G1 is 1.46 nm. Consequently, a feasible self-assembly mode of G1 gelators in water is proposed (Fig. 4d). As shown in Fig. 4d, G1 molecules were not distributed in a simple plane, but stacked with each other at a certain angle by π-π stacking force of the benzene rings. Then the fine nanofibers were formed. The XRD analysis showed that fine nanofibers aggregated model were based on hexagonal close packing. Further self-association of the one-dimensional fibers leads to rope-like fiber bundles with different sizes via VDW forces provided by long carbon chains. Eventually, a 3D network formed through intertwining of fiber bundles with the water confined.

## Conclusions

In conclusion, we have described a novel multi-functional gelator based on d-gluconic acetal-based derivatives, which exhibited highly efficient self-healing properties. We also provided a simple and effective method for regulation of thixotropy of LMWGs. Furthermore, the hydrogel formation mechanism was proposed based on the analysis of FT-IR, 1HNMR, and XRD. Further studies on the component structure–gel property relationship and exploring applications of these materials are still in progress.

## Additional file

Additional file 1: **Scheme S1.** The synthetic routes of Gn. **Table S1.** Gelation behavior of gelator G1,G2,G3 and G4 in various solvents. **Table S2.** CGCs of G1 hydrogel via Hofmeister anions and the viscosity B coefficients of Hofmeister sodium salts. **Table S3.** the effect of anions and Tgel of G1 hydrogel as a function of salt concentration. **Figure S1.** Oscillatory rheological study of hydrogel from G1 (2.5%, *w*/*v*) in in present of Hofmeister anions (concentration is 0.5 M): (a) Na_2_SO_4_, (b) Na_2_S_2_O_3_, (c) Na_2_HPO_4_, (d) NaF, (e) H_2_O, (f) NaCl, (g) NaBr, (h) NaNO_3_, (i) NaI, (j) NaClO_4_, (k) NaSCN at 25 °C, demonstrating high G’values (2.75 × 105, 2.69 × 105, 2.55 × 105, 3.50 × 105, 1.72 × 105, 2.34 × 105,2.25 × 105,2.14 × 105,1.55 × 105,2.50 × 105,1.1 × 104 Pa, respectively), flowing point (4.63%, 7.53%, 22.69%, 6.34%, 3.68%, 9.97%, 20.15%,3.51%,4.30%,13.88%,4.42%,respectively). The step-strain measurement shows the recovery ratios of G’ after the first cycle (90.74%, 85.93%, 82.08%, 100%, 90.77%, 100%, 95.56%,96.48%,95.97%,88.12%,93.89%respectively). **Figure S2.** (a) Frequency sweep of hydrogels from G1 (2.5%, *w*/*v*) with Hofmeister salts (concentration is 0.5 M) with a fixed strain (0.1%) at 25 °C; (b) Rheological data under oscillatory stress experiment on hydrogels from G1 (2.5%, w/v) with Hofmeister salts (concentration is 0.5 M) with a fixed frequency (1 Hz) at 25 °C; (c) Time scan tests under alternating strain of 0.1% and 100% of G1 (2.5%, w/v) with Hofmeister salts (concentration is 0.5 M) with a fixed frequency at 1 Hz at 25 °C. **Figure S3.** SEM images of G1 xerogel obtained from hydrogel (2.5% w/v) in present of Na_2_SO_4_ aqueous solution(concentration is 0.5 M); (b) SEM images of G1 xerogel obtained from hydrogel (2.5% w/v); (c) SEM images of G1 xerogel obtained from hydrogel (2.5% w/v) in present of NaCl aqueous solution(concentration is 0.5 M); (d) SEM images of G1 xerogel obtained from hydrogel (2.5% w/v) in presence of NaSCN aqueous solution (concentration is 0.5 M). **Figure S4.** The energy-minimized mode of G1. The length of molecular PG16 is 14.6 Å. (DOCX 3013 kb)

## Abbreviations

^1^HNMR: H nuclear magnetic resonance
CGCs: A minimum gel concentration
Co-Gels: Combined gel
FESEM: Field emission scanning electron microscope
FT-IR: Fourier transform infrared spectroscopy
LMMGs: Low molecular mass gelators
PXRD: Powder X-ray diffraction
T_gel_: The temperature of phase transition
