# Supplementary material for: Hofmeister Anion-Induced Tunable Rheology of Self-Healing Supramolecular Hydrogels
Source: Nanoscale Res Lett. 2019 Jan 7;14:5. doi: 10.1186/s11671-018-2823-8 (PMC6321834; doi:10.1186/s11671-018-2823-8)
Supplement: Supplementary file 1 — Scheme S1. The synthetic routes of Gn. Table S1. Gelation behavior of gelator G1,G2,G3 and G4 in various solvents. Table S2. CGCs of G1 hydrogel via Hofmeister anions and the viscosity B coefficients of Hofmeister sodium salts. Table S3. the effect of anions and Tgel of G1 hydrogel as a function of salt concentration. Figure S1. Oscillatory rheological study of hydrogel from G1 (2.5%, w/v) in in present of Hofmeister anions (concentration is 0.5 M): (a) Na2SO4, (b) Na2S2O3, (c) Na2HPO4, (d) NaF, (e) H2O, (f) NaCl, (g) NaBr, (h) NaNO3, (i) NaI, (j) NaClO4, (k) NaSCN at 25 °C, demonstrating high G’values (2.75 × 10^5^, 2.69 × 10^5^, 2.55 × 10^5^, 3.50 × 10^5^, 1.72 × 10^5^, 2.34 × 105,2.25 × 105,2.14 × 105,1.55 × 105,2.50 × 105,1.1 × 104 Pa, respectively), flowing point (4.63%, 7.53%, 22.69%, 6.34%, 3.68%, 9.97%, 20.15%,3.51%,4.30%,13.88%,4.42%,respectively). The step-strain measurement shows the recovery ratios of G’ after the first cycle (90.74%, 85.93%, 82.08%, 100%, 90.77%, 100%, 95.56%,96.48%,95.97%,88.12%,93.89%respectively). Figure S2. (a) Frequency sweep of hydrogels from G1 (2.5%, w/v) with Hofmeister salts (concentration is 0.5 M) with a fixed strain (0.1%) at 25 °C; (b) Rheological data under oscillatory stress experiment on hydrogels from G1 (2.5%, w/v) with Hofmeister salts (concentration is 0.5 M) with a fixed frequency (1 Hz) at 25 °C; (c) Time scan tests under alternating strain of 0.1% and 100% of G1 (2.5%, w/v) with Hofmeister salts (concentration is 0.5 M) with a fixed frequency at 1 Hz at 25 °C. Figure S3. SEM images of G1 xerogel obtained from hydrogel (2.5% w/v) in present of Na2SO4 aqueous solution(concentration is 0.5 M); (b) SEM images of G1 xerogel obtained from hydrogel (2.5% w/v); (c) SEM images of G1 xerogel obtained from hydrogel (2.5% w/v) in present of NaCl aqueous solution(concentration is 0.5 M); (d) SEM images of G1 xerogel obtained from hydrogel (2.5% w/v) in presence of NaSCN aqueous solution (concentration is 0.5 M). Figure S4. The energy-m [file 11671_2018_2823_MOESM1_ESM.docx]

**Supplementary Materials for**

Hofmeister Anions Tunable Rheology of Self-sealing Supramolecular Hydrogels

JING ZHANG ^1,2,3^, BAOHAO ZHANG ^1,2^ ,QIANG CHEN^3^ , Bao Zhang ^1,2^*and JIAN SONG ^1,2^*

^1^ School of Chemical Engineering and Technology ,Tianjin University,Tianjin300350, China; [zhangjing2014@tju.edu.cn](mailto:zhangjing2014@tju.edu.cn)

^2^ The Co-Innovation Center of Chemistry and Chemical Engineering of Tianjin, Tianjin 300072,China

^3^ Renai College of Tianjin University, Tianjin 301636, China

*****Correspondence: baozhang@tju.edu.cn ; songjian@tju.edu.cn; Tel.: +86-135-0215-0057

**1. Experimental details**

1.1 Synthetic routes of Gn:

Scheme S1. The synthetic routes of Gn

1.2 Synthetic details:

The synthesis and characterization of the precursors 2, 4-(3, 4-dichloro) benzylidene Methy-D-Gluconate(A) was reported previously.[1]

5 g (0.014 mol) 2, 4-(3, 4-dichloro) benzylidene Methy-D-Gluconate was dissolved in 50 mL methanol, then 2.19 g (0.021 mol) β-hydroxyethylene diamine and 0.01g DMAP (0.008 mmol) were added. The reaction mixture was stirred for 12h and then 20mL water was added. Subsequently，the white solid was collected by filtration. The filter cake was washed with water for twice and recrystallized with methanol to obtain compound G1 with a yield of 60%. Similarly, G2, G3 and B were obtained from 2,4-(3,4-dichloro) benzylidene Methy-D-Gluconate with 2,2-iminodi(ethylamine)，1-aminohexane and N-Propyl-propane-1,3-diamine respectively and purified by the same method.

5 g CH_3_I(0.033mol), 5g compound B（0.011mol）and 2.5g NaOH (0.06mol) were mixed and stirred for 8h at room temperature. Subsequently, the white solid was collected by filtration. The filter cake was washed with water for twice and recrystallized with methanol to obtain compound G4 with a yield of 55%.

1.3 Chemical characterization

G1：^1^H NMR(400MHz，DMSO-d6)：δ7.86(s，1H，CO-NH)，7. 68-7.66(d，1H，Ar-H)，7.55-7.52(d，1H，Ar-H)，7.48-7.45(t，1H，Ar-H)，5.68(s，1H，OCHO)，4.37-4.37(d，1H，OH)， 3.99(s，1H，CH)， 3.78-3.75(d，1H，CH_2_)，3.67(s，1H，CH)，3.57(m，1H，CH)，3.57-3.56(d，1H，CH_2_)，3.54(d，1H，CH_2_)，3.44-3.40(m，1H，CH2)，3.26(m，1H，NH)，3.15(m，1H，NH_2_)。

G2：1H NMR(400MHz，DMSO-d6)：δ7.87(s，1H，CO-NH)，7.70-7.65(d，1H，Ar-H)，7.57-7.53(d，1H，Ar-H)，7.45-7.51(t，1H，Ar-H)，5.68(s，1H，OCHO)，4.78-4.83(m，1H，NH)，4.75-4.71(d，1H，OH)，4.47-4.43(d，2H，OH)，4.37(s，1H，CH)，4.00(s，1H，OH)，3.77(d，1H，CH_2_)，3.65(s，1H，CH)，3.55(m，1H，CH)，3.45-3.41(d，1H，CH_2_),3.25-3.20(d，1H，CH_2_)，2.60-2.54(m，1H，CH_2_)。

G3：1H NMR (400 MHz, DMSO): δ7.90 (s, 1H，CO-NH), 7.67 (d, 1H，Ar-H), 7.60-7.58 (d，1H，Ar-H), 7.47-7.44(t，1H，Ar-H)，5.67 (s，1H，OCHO), 5.10 (m，1H，NH), 4.35 (d，1H，OH), 4.00 (s，1H，CH), 3.76(d，1H，CH_2_), 3.75-3.53 (m，1H，CH), 3.50 (d，1H，CH_2_), 3.47-3.10 (m，1H，CH_2_), 3.10-1.25 (m，1H，CH), 3.05-1.25 (m，1H，CH), 1.85-1.51 (m，1H，CH),1.51-1.26 (t，3H，CH_3_).

G4：1H NMR (400 MHz，DMSO) : δ 7.94-7.84 (s，1H，CONH)，7.71-7.61 (d，1H，Ar-H)，7.61-7.51 (d，1H，Ar-H)，7.51-7.36 (t，1H，Ar-H)，5.71-5.62 (s，1H，OCHO)，4.79-4.62(s，2H，OH)，4.39-4.29 (s， 1H，CH_2_)，4.04-3.96(d，1H，CH)，3.83-3.71(m，1H，CH)，3.69-3.59(m，1H， CH_2_)，3.59-3.49(m，1H，CH)，3.47-3.40(m，1H，CH)，3.19-3.03(m，2H，CH_2_)， 1.50-1.35(d，2H，CH_2_)，1.34-1.12(s，6H，CH_2_)，0.96-0.76 (t，3H，CH_3_)。

**2. Additional data**

| Table S1 Gelation behavior of gelator G1,G2,G3 and G4 in various solvents | | | | |
| --- | --- | --- | --- | --- |
| sovent | G1 | G2 | G3 | G4 |
| n-propyl alcohol | P | P | S | OG |
| n-octyl alcohol | OG | OG | PG | OG |
| isooctyl alcohol | OG | OG | PG | OG |
| toluene | P | P | TG | OG |
| o-dichlorobenzene | OG | OG | TG | OG |
| n-hexane | I | I | I | I |
| acetone | I | I | PS | PG |
| chloroform | P | P | TG | PG |
| acetonitrile | I | P | PG | PG |
| 1,4-dioxane | P | P | S | S |
| DMSO | S | S | S | S |
| NMP | S | S | S | S |
| THF | I | I | OG | S |
| butyl acetate | TG | I | TG | OG |
| water | OG^(0.17,45.9)^ | OG | P | P |

Gel Concentration: 2.5 % (w/v). OG: opaque gel; TG: Transparent gel; P: precipitate; S: solution. I: Insoluble.

Table S2 CGCs of G1 hydrogel via Hofmeister anions and the viscosity B coefficients of Hofmeister sodium salts.

|  | CGCs  % (w/v) | the viscosity B  coefficients |
| --- | --- | --- |
| SO_4_^2﹣^ | 1.282 | 0.206 |
| S_2_O_3_^2-^ | 1.307 | 0.014 |
| HPO_4_^2-^ | 1.389 | 0.340 |
| F^﹣^ | 1.869 | 0.127 |
| Cl^﹣^ | 2.128 | -0.005 |
| Br^﹣^ | 2.439 | -0.033 |
| NO_3_^﹣^ | 2.439 | -0.043 |
| I^﹣^ | 2.778 | -0.073 |
| H_2_O | 2.500 | 0 |
| ClO_4_^﹣^ | 1.852 | -0.060 |
| SCN^﹣^ | 3.125 | -0.103 |

Note: salts Concentration: 0.5M.

Table S3 the effect of anions and T_gel_ of G1 hydrogel as a function of salt concentration

| C(mol∙L^-1^) | SO_4_^2﹣^  T_gel_  (℃) | S_2_O_3_^2-^  T_gel_  (℃) | HPO_4_^2-^  T_gel_  (℃) | F^-^  T_gel_  (℃) | Cl^﹣^  T_gel_  (℃) | Br^﹣^  T_gel_  (℃) | NO_3_^﹣^T_gel_  (℃) | I^﹣^  T_gel_  (℃) | ClO**_4_^﹣^**  T_gel_  (℃) | SCN^﹣^  T_gel_  (℃) |
| --- | --- | --- | --- | --- | --- | --- | --- | --- | --- | --- |
| 0 | 45.9 | 45.9 | 45.9 | 45.9 | 45.9 | 45.9 | 45.9 | 45.9 | 45.9 | 45.9 |
| 0.1 | 56.8 | 55.1 | 54.6 | 54.0 | 52.6 | 51.4 | 51.4 | 74.6 | 66.7 | 50.1 |
| 0.3 | 69.1 | 68.8 | 68.5 | 58.9 | 55.7 | 54.7 | 54.8 | 78.8 | 74.2 | 52.8 |
| 0.5 | 72.0 | 70.9 | 70.9 | 60.2 | 57.1 | 55.8 | 55.6 | 79.4 | 75.7 | 54.6 |
| 0.7 | 73.8 | 74.0 | 72.8 | 63.8 | 59.5 | 57.3 | 56.3 | 83.8 | 76.9 | 56.9 |
| 1 | 75.4 | 75.2 | 74.2 | 65.9 | 64.2 | 63.3 | 63.2 | PG | 77.8 | PG |
| 2 | P | 76.7 | 75.7 | -- | 78.2 | 70.1 | 65.5 | PG | PG | PG |
| 3 | P | P | 79.9 | -- | 81.8 | 73.2 | 70.7 | PG | PG | PG |
| 4 | P | P | 85.3 | -- | 83.1 | PG | 73.2 | PG | PG | PG |
| 5 | P | P | 88.9 | -- | 86.0 | PG | 75.1 | PG | PG | PG |

Notes：P： Precipitate； PG： Partial gel；the gelator concentration：0.025g·mL^-1^

Rheological Study


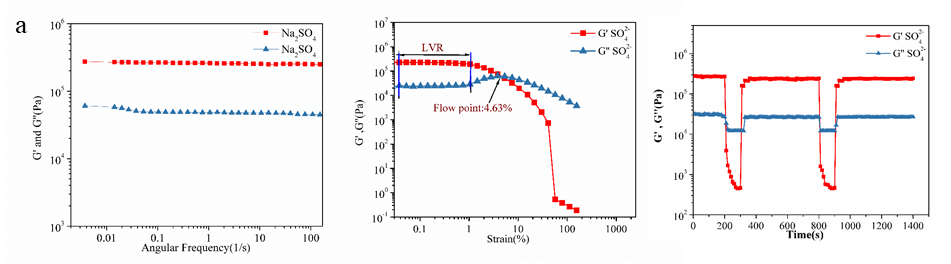


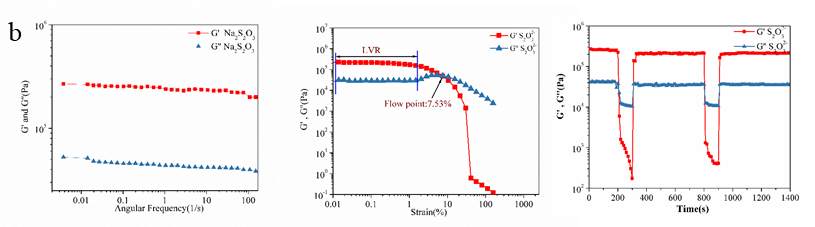


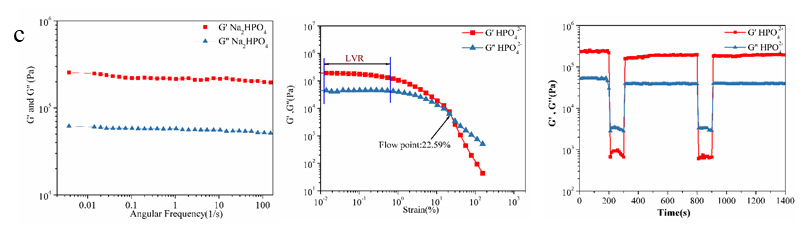


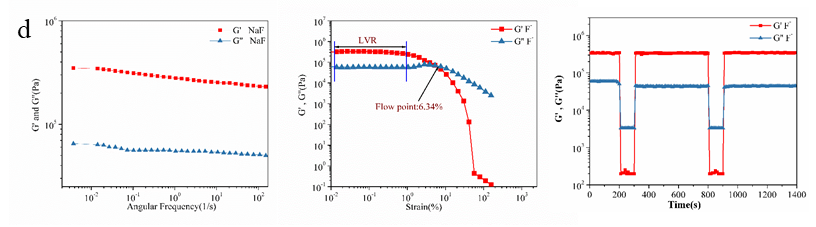


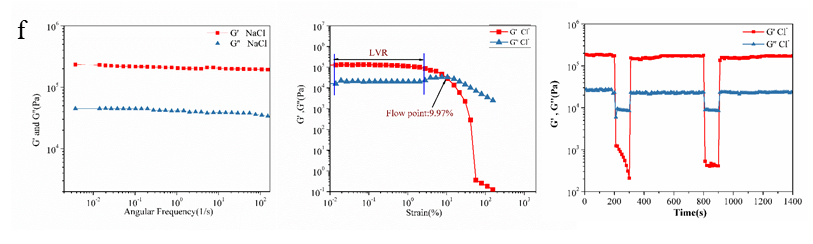


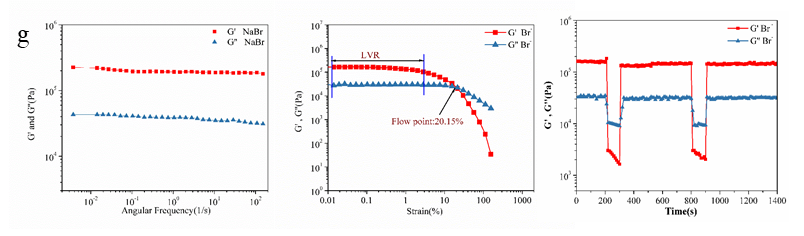


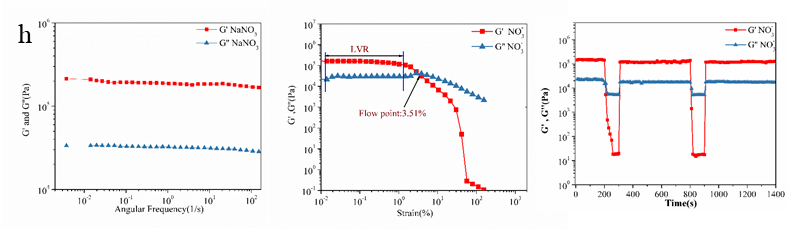


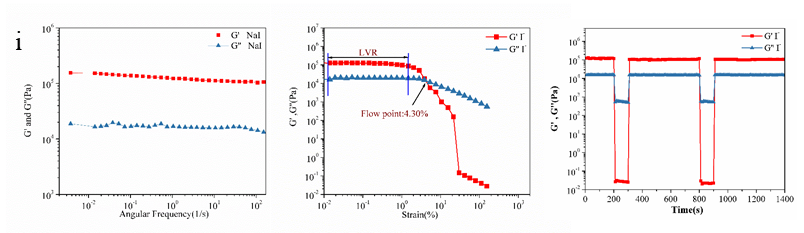


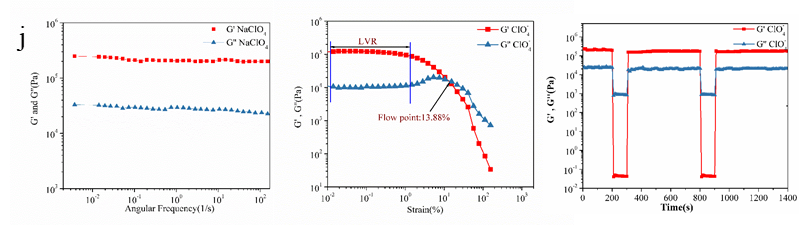


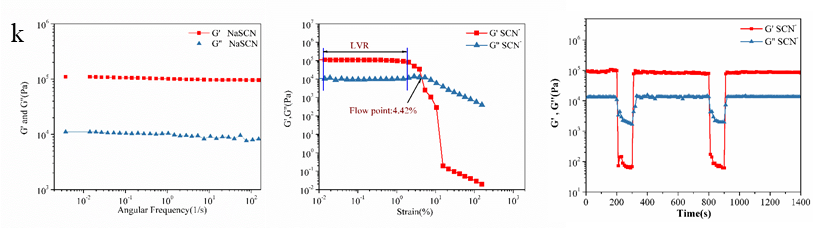


Figure S1. Oscillatory rheological study of hydrogel from G1 (2.5%, w/v) in in present of Hofmister anions (concentration is 0.5M) :(a) Na_2_SO_4_, (b) Na_2_S_2_O_3_, (c) Na_2_HPO_4_, (d) NaF, (e) H_2_O, (f) NaCl ,(g) NaBr, (h) NaNO_3_,(i) NaI, (j) NaClO_4_, (k) NaSCN at 25℃, demonstrating high G’values (2.75×10^5^, 2.69×10^5^, 2.55×10^5^, 3.50×10^5^, 1.72×10^5^, 2.34×10^5^，2.25×10^5^，2.14×10^5^，1.55×10^5^，2.50×10^5^，1.1×10^4^Pa, respectively), flowing point (4.63%, 7.53%, 22.69%, 6.34%, 3.68%, 9.97%, 20.15%，3.51%，4.30%，13.88%，4.42%，respectively). The step-strain measurement shows the recovery ratios of G’ after the first cycle (90.74%, 85.93%, 82.08%, 100%, 90.77%, 100%, 95.56%，96.48%，95.97%，88.12%，93.89%respectively)


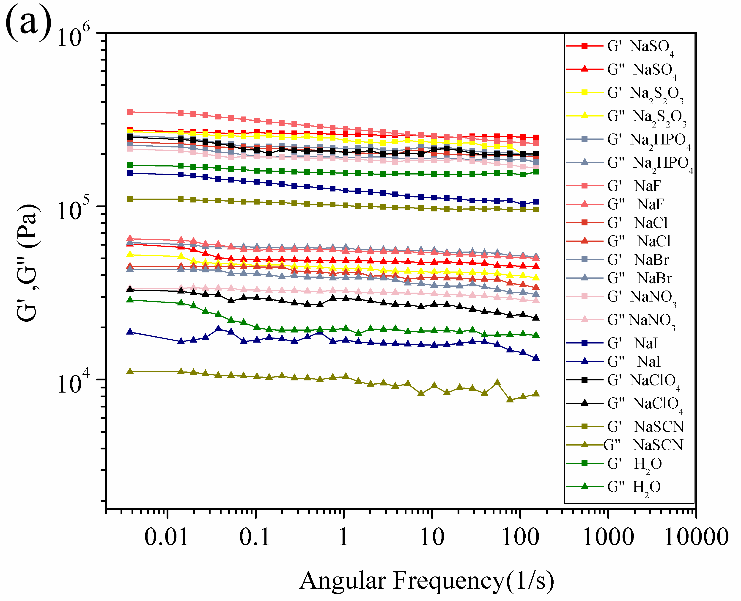


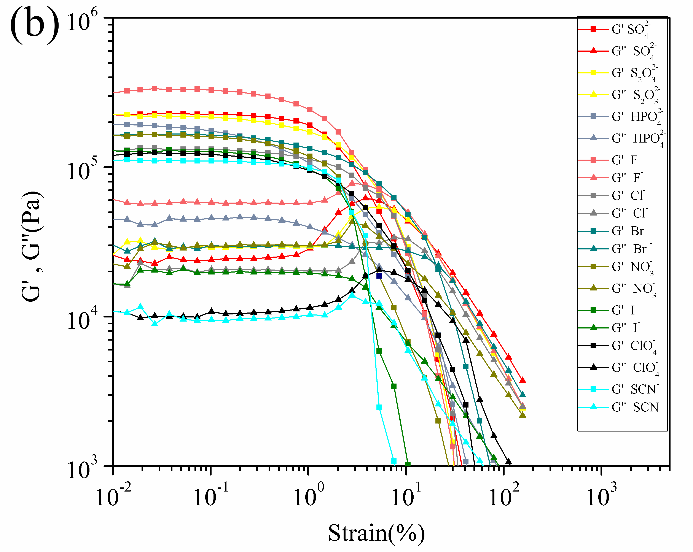


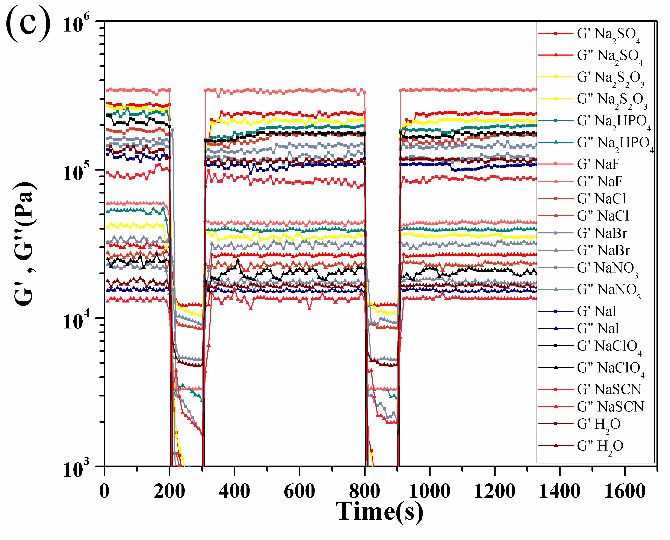


Figure S2 (a) Frequency sweep of hydrogels from G1 (2.5%, w/v) with Hofmeister salts (concentration is 0.5M) with a fixed strain (0.1%) at 25℃; (b) Rheological data under oscillatory stress experiment on hydrogels from G1 (2.5%, w/v) with Hofmeister salts (concentration is 0.5M) with a fixed frequency (1 Hz) at 25℃; (c) Time scan tests under alternating strain of 0.1% and 100% of G1 (2.5%, w/v) with Hofmeister salts (concentration is 0.5M) with a fixed frequency at 1 Hz at 25℃.

SEM


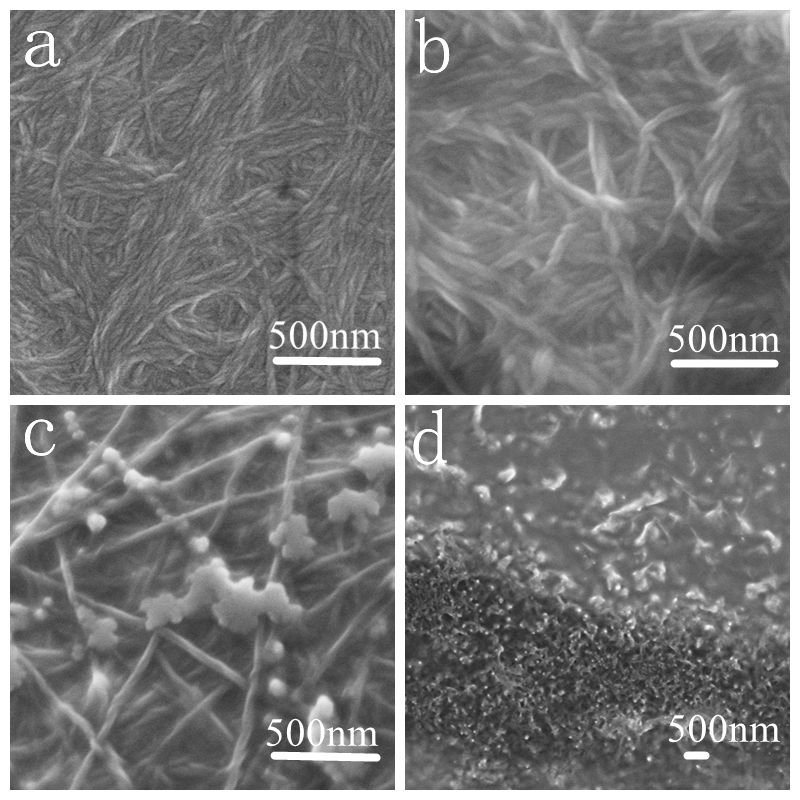


Figure S3: SEM images of G1 xerogel obtained from hydrogel (2.5% w/v) in present of Na_2_SO_4_ aqueous solution(concentration is 0.5M); (b) SEM images of G1 xerogel obtained from hydrogel (2.5% w/v); (c) SEM images of G1 xerogel obtained from hydrogel (2.5% w/v) in present of NaCl aqueous solution(concentration is 0.5M) ; (d) SEM images of G1 xerogel obtained from hydrogel (2.5% w/v) in present of NaSCN aqueous solution(concentration is 0.5M).


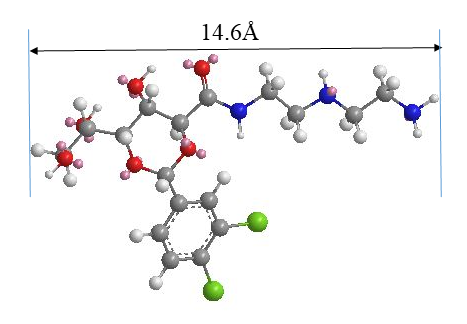


Figure S4. The energy-minimized mode of G1. The length of molecular PG16 is 14.6Å

References:

1. a) Xidong Guan, Kaiqi Fan, Tongyang Gao, Anping Ma, Bao Zhang and Jian Song. Chem. Commun., 2016, 52, 962-965; b) Shipeng Chen, Baohao Zhang, Nanxiang Zhang, Fengsheng Ge, Bao Zhang,Xiaoji Wang, and Jian Song, ACS Appl. Mater. Interfaces 2018, 10, 5871−5879.
